# An Interactive Mobile App Game to Address Aggression (RegnaTales): Pilot Quantitative Study

**DOI:** 10.2196/13242

**Published:** 2019-05-08

**Authors:** Jeffrey G Ong, Nikki S Lim-Ashworth, Yoon P Ooi, Jillian S Boon, Rebecca P Ang, Dion H Goh, Say H Ong, Daniel S Fung

**Affiliations:** 1 Department of Developmental Psychiatry Institute of Mental Health Singapore Singapore; 2 Department of Psychology University of Basel Basel Switzerland; 3 Psychological Studies National Institute of Education Nanyang Technological University Singapore Singapore; 4 Wee Kim Wee School of Communication and Information Nanyang Technological University Singapore Singapore

**Keywords:** video games, mental health, anger management, mobile app

## Abstract

**Background:**

The rapid advancement in media technology has radically changed the way we learn and interact with one another. Games, with their engaging and interactive approach, hold promise in the delivery of knowledge and building of skills. This has potential in child and adolescent mental health work, where the lack of insight and motivation for therapy are major barriers to treatment. However, research on the use of serious games in mental health interventions for children and adolescents is still in its infancy.

**Objective:**

This study adds to the research on serious games in mental health interventions through the development and evaluation of RegnaTales, a series of 6 mobile apps designed to help children and adolescents manage anger. We examined the usability and playability of RegnaTales, as well as children’s aggression levels before and after the game play.

**Methods:**

A total of 72 children aged between 6 and 12 years were recruited for the study. Thirty-five participants had a clinical diagnosis of disruptive behavior disorders (DBD), whereas 37 were typically developing (TD) children. Each child played 1 of the 6 RegnaTales apps for approximately 50 min before completing the Playability and Usability Questionnaire. The Reactive-Proactive Aggression Questionnaire was completed before and after the game play.

**Results:**

The overall results showed high levels of enjoyment and playability. TD children and children with DBD had similar experienced fun and perceived playability scores on all 6 mobile apps. All 6 mobile apps garnered comparable experienced fun and perceived playability scores. Furthermore, 42% (5/12) to 67% (8/12) of the children indicated that they would like to play the games again. Importantly, children felt that they acquired skills in anger management, were motivated to use them in their daily lives, and felt confident that the skills would help them better manage their anger. Children reported significantly lower reactive aggression after playing the mobile apps Rage Raver (*P*=.001), Abaddon (*P*=.008), and RegnaTools (*P*=.03). These apps focused on the psychoeducation of the link between thoughts and emotions, as well as equipping the participants with various emotion regulation strategies such as relaxation and cognitive restructuring.

**Conclusions:**

This study presents evidence to support RegnaTales as a feasible serious game. The preliminary findings associated with reduction in reactive aggression, coupled with future research to further establish its efficacy, could warrant RegnaTales as a potential intervention for anger issues among clinical and community populations.

## Introduction

### Background

Anger issues are among one of the most frequent reasons for referral to intervention within the developmental population [1]. A significant number of childhood mental health problems, particularly attention-deficit/hyperactivity disorder (ADHD; prevalence of 5%), conduct disorder (CD; prevalence of 4%), and oppositional defiant disorder (ODD; prevalence of 3.3%), have features associated with anger, including irritability and aggression toward others [2]. Evidence suggests that distorted thinking patterns often cause psychosocial interactions to be appraised in a maladaptive manner, triggering anger, aggression, and disruptive behavior [3,4]. To address these cognitive biases, treatment needs to support children in restructuring existing unhelpful thought patterns and equip them with more adaptive response strategies [5].

Cognitive behavioral therapy (CBT) is an approach centered on the conceptual principles outlined above. It has been shown to be efficacious in the treatment of childhood anger problems [6,7]. However, CBT is typically administered through individual and group formats, which could be time-consuming and resource-intensive. Furthermore, parents are often required to accompany the child to see the therapist and this limits the accessibility of treatment [8,9]. Finally, children referred for anger management may not have insight and motivation for change, which are additional barriers for therapy [10]. With advancements in technology, there have been attempts to increase access to therapy through alternatives or adjuncts (eg, computers, internet, and mobile phones) to clinic-based therapy [11-18]. CBT, in particular, is well suited for such adaptations because of its structured and systematic format. A review of the literature indicated that where proven techniques are adapted for computer delivery, clinical outcomes are comparable with traditional face-to-face services [19,20].

### Serious Games Using Cognitive Behavioral Therapy Principles

Games hold promise in building skills and imparting knowledge to children as they find the interactive features, simulations, and immersive environments both enticing and engaging [21]. The game world is known to be a *safe* environment to practice behaviors in role-play situations through rule learning and repetition of tasks [22], and it has been demonstrated that games can improve skills in areas such as communication and problem-solving [23]. The use of games for learning, teaching, and psychological interventions is gaining attention. Serious games are defined as *entertaining games with non-entertainment goals* [24], and such games have adopted the CBT approach to address behavioral and emotional problems in children and adolescents [25,26].

### RegnaTales

RegnaTales, a series of 6 mobile apps, was created in response to the above advantages of using mobile technology and serious games to increase access to treatment and enhance the motivation of children to learn skills [27,28]. RegnaTales was developed from the Social Problem-Solving Skills Training (SPSST) for anger management [29], based on a cognitive behavioral framework. The SPSST was originally administered in a face-to-face therapy setting through a workbook. It was subsequently developed into a Web-based social problem-solving game, Socialdrome [30] and evolved into the current interactive role-playing game–based mobile apps—RegnaTales. See Multimedia Appendix 1 for screenshots of the RegnaTales mobile apps.

The first 4 mobile apps are game-based apps that teach various anger management strategies, whereas the fifth and sixth apps focus on helping the children apply these skills in their daily lives. The content of each mobile app is listed in Table 1.

**Table 1:** List of RegnaTales apps.

RegnaTales apps		Objectives
**Game-based mobile apps**		
	Village of Lost Expressions	Learn to identify different feelings
	Rage Raver	Learn to distinguish between positive and negative thoughts; learn to identify bodily signs of anger; learn to replace negative thoughts with positive thoughts; learn deep breathing; learn guided imagery
	The Illusionist	Learn perspective taking; use of cognitive restructuring techniques; use of deep breathing; use of guided imagery; exposure to a variety of leisure activities to cope with anger
	Abaddon	Fighting fair; being firm; use of perspective taking; use of cognitive restructuring techniques; use of deep breathing; use of guided imagery; use of leisure activities to cope with anger
**Therapeutic tools**		
	RegnaTools	Anger coping skills
	TimeOut!	Daily monitoring of feelings and intensity; increase awareness of physiological symptoms; log of triggering events; trigger use of anger coping strategies when angry; feedback on the frequency of each feelings throughout the week; monitor use of anger coping strategies

In a pilot evaluation of the first RegnaTales mobile game app by Ooi et al [31] conducted in Switzerland, the children found the game highly playable and enjoyable. However, this evaluation was limited in scope because only 1 of the apps was utilized, the number of participants was small, and it was done with a nonclinical population.

The aim of this study was to evaluate RegnaTales in the context of the apps’ usability and playability across domains, including experienced fun, perceived playability, understanding of goals, and perceived impact. In addition, our study explored whether RegnaTales helped in attenuating aggression.

## Methods

### Recruitment

Recruitment took place from November 2015 to December 2017. Participants with a disruptive behavior disorders (DBD) diagnosis were recruited from a psychiatric outpatient clinic in Singapore. According to the Diagnostic and Statistical Manual of Mental Disorders, fourth edition, text revision [32], DBD is a cluster of childhood mental health diagnoses that includes ADHD, ODD, and CD. Flyers were put up in the community to recruit children who have a typically developing (TD) profile.

A total of 72 children aged between 6 and 12 years, comprising those with a clinical diagnosis of DBD and those who were TD, participated in the study. A total of 68 children with DBD were referred for the study by their psychiatrists, of which 35 agreed to participate. The majority of these participants were diagnosed with ADHD only (n=33), whereas 1 had a comorbid diagnosis of ODD and ADHD, and the remaining participant had a diagnosis of CD and ADHD. Another 37 children with a TD profile responded to flyers put up in the community and were recruited for the study. The study was approved by the Domain Specific Review Board (DSRB) of the National Healthcare Group (DSRB 2015/00986). Written informed consent and assent were obtained from the child participants and their parents before commencement of the study procedures.

### Procedure

The 6 RegnaTales apps were developed sequentially, with each app taking about 4 months to develop. Upon completing the development of each app, 12 child participants were recruited to test the app. We tried to recruit an equal number of TD children and children with DBD to test each app. Each RegnaTales app was tested by a different group of 12 child participants. The children played the assigned RegnaTales app on an Apple iPad (9.7-inch screen size) for approximately 50 min before completing the Playability and Usability Questionnaire (PUQ) [31]. They also completed the Reactive-Proactive Aggression Questionnaire [33] before and after the game play. The aggression scores of 2 children using the Abaddon mobile app were not captured because of a technical problem during the research study (ie, data were not captured by the server). The same procedure was followed for each RegnaTales app. Each participant was reimbursed Singapore Dollar $30 at the end of the visit.

### Technical Details

RegnaTales was developed using the Corona development platform for mobile apps with the back end running PHP and a MySQL database on a Microsoft Windows Server. RegnaTales has the capability of being ported to various mobile operating systems and devices. Character animation and user interaction were accomplished using Kwik, an Adobe Photoshop plugin. RegnaTales is delivered using the English language.

### Measures

#### Playability and Usability Questionnaire

Similar to the study by Ooi et al [31], elements of the gaming experience such as fun, game experience, willingness to continue playing, and curiosity were measured using the following 4 domains from the extended Short Feedback Questionnaire (eSFQ) [34]:

- Experienced fun: This was measured using a 5-point rating scale, where 1=*yawn, boring* and 5=*yeah, fun*
- Game experience: This was assessed by getting children to mark predefined attributes (ie, *simple, difficult, great, childish, fun, boring, exciting, tiring, intuitive* and *confusing*)
- Willingness to continue using the game: This was assessed using the question *Would you like to play the game again?* answered with *yes, maybe,* or *no*
- Curiosity: This was measured with 2 items on a 5-point Likert scale where 1=*completely disagree* to 5=*completely agree*

In addition to the above items from the eSFQ, the PUQ included the following 2 domains to further understand the experience and understanding of the children:

- Perceived playability: Perceived playability of the mobile apps was measured using 9 items from Tan et al [35] (5-point Likert scale where 1=*completely disagree* to 5=*completely agree*)

- Understanding of the goals of the game (6 multiple choice options)

As mobile apps 5 and 6 are nongame-based and are focused on the application of skills, some irrelevant items (eg, whether they liked the story and what they thought were the goals of the games) were removed and an additional domain to assess the knowledge, attitudes, and intentions related to the target behavior of anger management was added. This is a modification of 5 items based on the user version of the Mobile Application Rating Scale [36]:

- Perceived impact (5 items, 5-point Likert scale where 1=*completely disagree* to 5=*completely agree*).

#### Reactive-Proactive Aggression Questionnaire

This is a brief self-report scale that comprises 23 items measuring reactive (11 items, Cronbach alpha=.83) and proactive (12 items, Cronbach alpha=.82) aggression among children and yields an overall score of aggression. Items are rated on a 3-point Likert scale (0=*never*, 1=*sometimes*, and 2=*often*). The Reactive-Proactive Aggression Questionnaire (RPQ) has been validated extensively for use with the developmental population in reporting aggression and has been shown to have robust psychometric properties [37,38].

### Data Analysis

Independent sample *t* tests (2-tailed) were used to compare the aggression levels of children with DBD with that of TD children, before using the mobile apps. To understand the participants’ experience of RegnaTales, descriptive statistics were used to present the playability and usability feedback. Mann-Whitney tests (2-tailed) were conducted to examine the differences between children with DBD and TD children in terms of age, playability, and usability scores. Kruskal-Wallis *H* tests were conducted to examine if there were differences in playability and usability scores among the mobile apps. In addition, Wilcoxon signed rank tests (2-tailed) were conducted to examine if there were changes in aggression after game play. All statistical significance levels were determined at *P*<.05.

## Results

### Overview

There was a total of 23 female and 49 male participants, aged between 6 and 12 years, with a mean age of 8.79 (SD 1.73) years. Table 2 presents the demographics of the participants for each of the 6 mobile apps. Mann-Whitney tests showed that there were no significant differences in the ages of children in the DBD group and children in the TD group across all 6 mobile apps.

Table 3 shows the RPQ scores of TD children and children with DBD before game play. Independent sample *t* tests showed that children with DBD had higher reactive aggression, with *t*_70_=−2.60 and *P*=.01; proactive aggression, with *t*_70_=−3.58 and *P*=.001; and overall aggression, with *t*_70_=−3.53 and *P*=.001, on the RPQ as compared with TD children before using the mobile apps.

### Experienced Fun

As shown in Table 4, experienced fun scores ranged from 4.17 (Rage Raver) to 4.67 (RegnaTools). A Kruskal-Wallis *H* test showed that there were no significant differences in the experienced fun scores across the 6 mobile apps, with χ^2^_5_=0.5 and *P*=.99. Mann-Whitney tests indicated that there were no significant differences in the experienced fun scores between TD children and children with DBD in each of the 6 mobile apps (see Table 4).

### Game Experience

**Figure 1:**
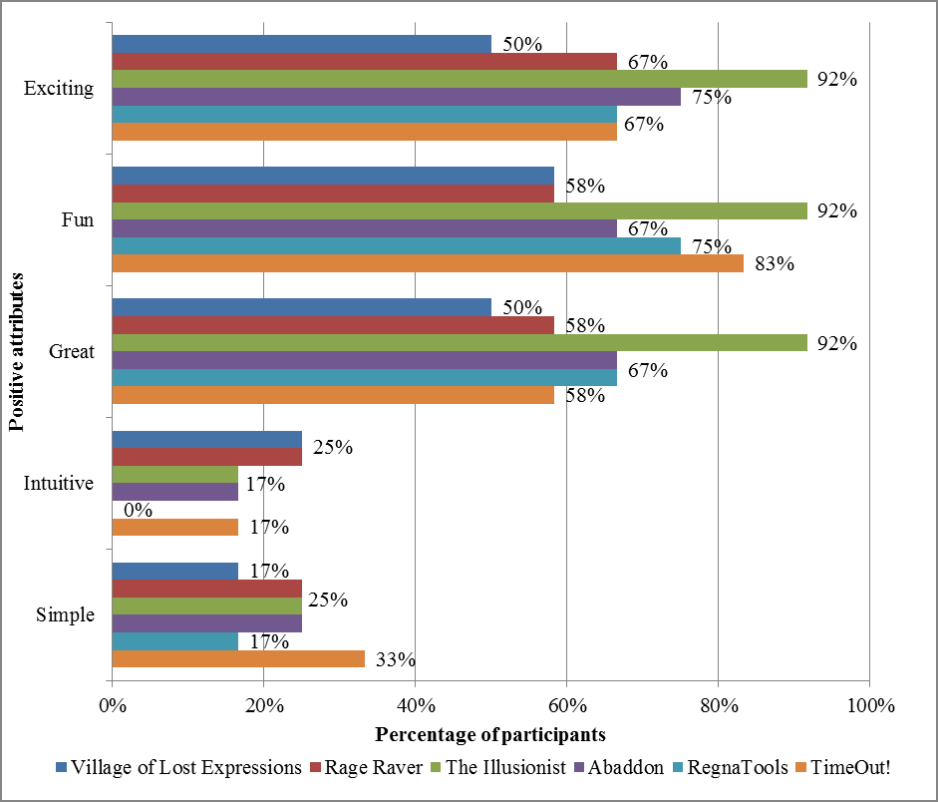
Positive attributes of the RegnaTales mobile apps.

**Figure 2:**
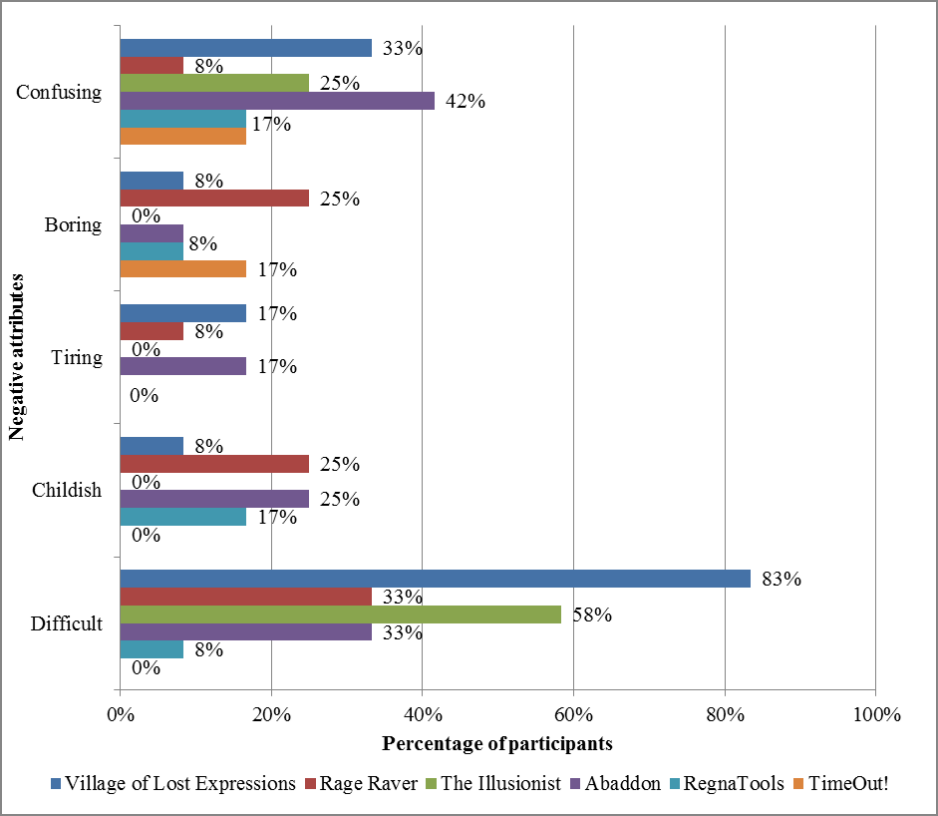
Negative attributes of the RegnaTales mobile apps.

To better understand the participants’ experience of the mobile apps, we looked at the attributes selected by the participants in describing their experience when using each of the 6 mobile apps (Figures 1 and 2).

Generally, half or more participants found the mobile apps *exciting*, *fun*, and *great*. However, less than one-third of them found the apps *intuitive* and *simple*.

Figure 2 shows that the mobile apps differed vastly in their difficulty levels. For Village of Lost Expressions and The Illusionist, more than half of the participants found the tasks difficult. However, on the other 4 mobile apps, most participants did not find the tasks difficult.

### Continued Usage

**Figure 3:**
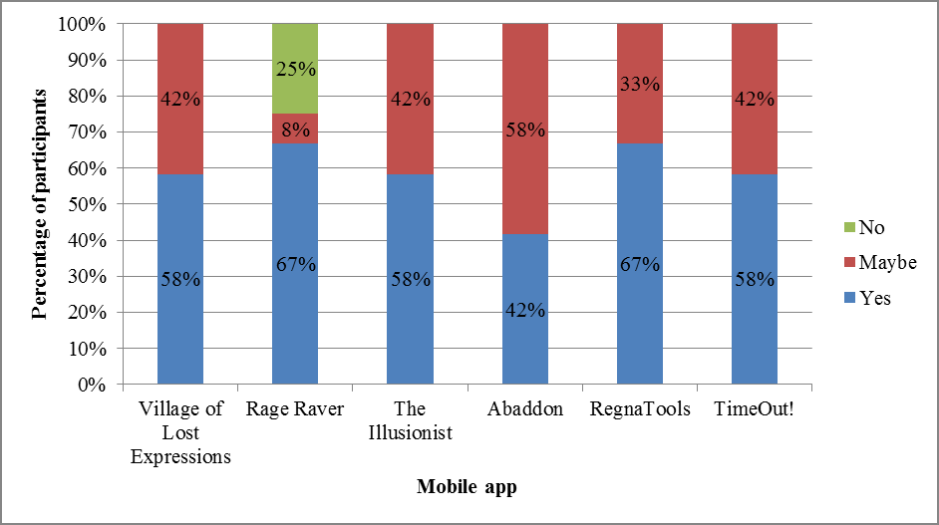
Continued usage for mobile apps.

Figure 3 shows the participants’ responses when asked if they would like to play the various mobile apps again.

With the exception of Rage Raver, all participants indicated either *yes* or *maybe*, when asked if they would like to play the apps again and none of the participants indicated *no*.

### Curiosity

As shown in Table 5, curiosity scores ranged from 4.04 (Rage Raver) to 4.54 (The Illusionist). A Kruskal-Wallis *H* test showed that there were no significant differences in the curiosity scores among the 4 mobile game apps, with χ^2^_3_=2.4 and *P*=.51. Overall, 38 out of 48 (79%) participants indicated that they wanted to see more of the game world, whereas 41 out of 48 (86%) indicated that they were curious to see what would happen next in the game. Mann-Whitney tests indicated that there were no significant differences in the curiosity scores between TD children and children with DBD in each of the 4 mobile game apps (see Table 5).

### Perceived Playability

As shown in Table 6, perceived playability scores ranged from 3.91 (Rage Raver) to 4.33 (The Illusionist). A Kruskal-Wallis *H* test showed that there were no significant differences in the perceived playability scores among the 6 mobile apps, with χ^2^_5_=6.3 and *P*=.28. Mann-Whitney tests indicated that there were no significant differences in the perceived playability scores between TD children and children with DBD in each of the 6 mobile apps, as shown in Table 6.

**Figure 4:**
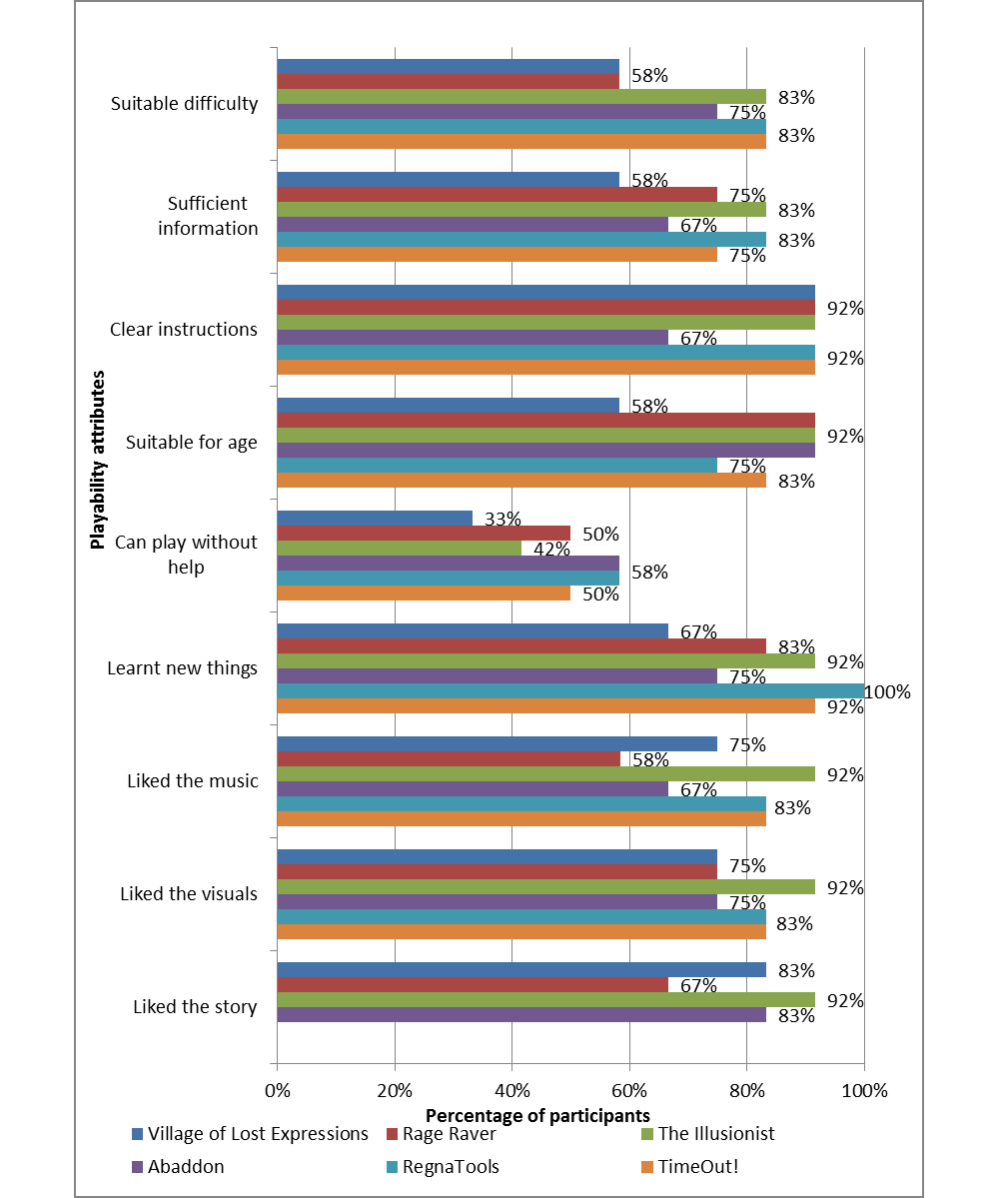
Perceived playability for mobile apps.

As shown in Figure 4, more than 50% (6/12) of children agreed/strongly agreed that RegnaTales had good playability features on all domains, with the exception of *can play without help*, where 33% (4/12) to 58% (7/12) agreed/strongly agreed.

### Understanding of the Goals of the Game

The children indicated the goals of the game apps as the following: save the hero’s parents (39 out of 48, 81%), make friends (19 out of 48, 40%), gain more points and power (21 out of 48, 44%), learn to recognize other’s feelings (34 out of 48, 71%), and learn more about themselves (28 out of 48, 58%).

### Perceived Impact

As shown in Table 7, perceived impact score was 4.40 for RegnaTools and 4.45 for TimeOut!. Mann-Whitney tests indicated that there were no significant differences in the perceived impact scores between children who used RegnaTools and TimeOut!, with *U*=64.0 and *P*=.66.

**Table 2:** Demographics of participants for each mobile app.

Demographics				Typically developing (n=6)		Disruptive behavior disorder (n=6)		Total sample (n=12)
**Village of Lost Expressions**								
	**Gender, n**							
		Female		1		1		2
		Male		5		5		10
	Age (years), mean (SD)		8.17 (1.72)		8.5 (1.52)		8.33 (1.56)	
**Rage Raver**								
	**Gender, n**							
		Female		2		2		4
		Male		4		4		8
	Age (years), mean (SD)		8.67 (2.16)		10 (1.67)		9.33 (1.97)	
**The Illusionist^a^**								
	**Gender, n**							
		Female		5		0		5
		Male		2		5		7
	Age (years), mean (SD)		8.29 (1.6)		9 (1.41)		8.58 (1.51)	
**Abaddon**								
	**Gender, n**							
		Female		3		0		3
		Male		3		6		9
	Age (years), mean (SD)		8 (2.28)		9 (1.21)		9.17 (2.12)	
**RegnaTools**								
	**Gender, n**							
		Female		3		0		3
		Male		3		6		9
	Age (years), mean (SD)		8.33 (1.97)		9.33 (1.37)		8.83 (1.7)	
**TimeOut!**								
	**Gender, n**							
		Female		6		0		6
		Male		0		6		6
	Age (years), mean (SD)		8.33 (1.86)		8.67 (1.37)		8.5 (1.57)	

^a^Typically developing (n=7); disruptive behavior disorder (n=5).

**Table 3:** Overall Reactive-Proactive Aggression Questionnaire scores before game play.

Category	Reactive aggression, mean (SD)	Proactive aggression, mean (SD)	Overall aggression, mean (SD)
Typically developing	0.68 (0.38)	0.11 (0.18)	0.38 (0.24)
Disruptive behavior disorder	0.92 (0.39)	0.32 (0.32)	0.61 (0.30)

**Table 4:** Experienced fun scores.

App name and partipicant category		Mean (SD)	*U* value	*P* value
**Village of Lost Expressions**			15	.99
	Typically developing (n=6)	4.67 (0.82)		
	Disruptive behavior disorder (n=6)	4.33 (1.03)		
	Total sample (n=12)	4.50 (0.90)		
**Rage Raver**			9	.18
	Typically developing (n=6)	5 (0)		
	Disruptive behavior disorder (n=6)	3.33 (1.97)		
	Total sample (n=12)	4.17 (1.59)		
**The Illusionist**			13	.52
	Typically developing (n=7)	4.71 (0.76)		
	Disruptive behavior disorder (n=5)	4.20 (1.10)		
	Total sample (n=12)	4.50 (0.90)		
**Abaddon**			14.5	.73
	Typically developing (n=6)	4.67 (0.82)		
	Disruptive behavior disorder (n=6)	4.00 (1.67)		
	Total sample (n=12)	4.33 (1.3)		
**RegnaTools**			18	.99
	Typically developing (n=6)	4.67 (0.82)		
	Disruptive behavior disorder (n=6)	4.67 (0.82)		
	Total sample (n=12)	4.67 (0.78)		
**TimeOut!**			15	.99
	Typically developing (n=6)	4.33 (1.03)		
	Disruptive behavior disorder (n=6)	4.67 (0.82)		
	Total sample (n=12)	4.50 (0.90)		

**Table 5:** Curiosity scores.

App name and partipicant category		Mean (SD)	*U* value	*P* value
**Village of Lost Expressions**			16	.87
	Typically developing (n=6)	4.25 (0.88)		
	Disruptive behavior disorder (n=6)	4.42 (0.66)		
	Total sample (n=12)	4.33 (0.75)		
**Rage Raver**			15.5	.99
	Typically developing (n=6)	4.33 (1.63)		
	Disruptive behavior disorder (n=6)	3.75 (1.94)		
	Total sample (n=12)	4.04 (1.74)		
**The Illusionist**			11	.41
	Typically developing (n=7)	4.86 (0.24)		
	Disruptive behavior disorder (n=5)	4.10 (1.47)		
	Total sample (n=12)	4.54 (0.99)		
**Abaddon**			10	.17
	Typically developing (n=6)	4.33 (1.21)		
	Disruptive behavior disorder (n=6)	4.17 (0.41)		
	Total sample (n=12)	4.25 (0.87)		

**Table 6:** Perceived playability scores.

App name and partipicant category		Mean (SD)	*U* value	*P* value
**Village of Lost Expressions**			14.5	.62
	Typically developing (n=6)	3.89 (0.53)		
	Disruptive behavior disorder (n=6)	4.00 (0.72)		
	Total sample (n=12)	3.94 (0.60)		
**Rage Raver**			17	.92
	Typically developing (n=6)	3.94 (0.97)		
	Disruptive behavior disorder (n=6)	3.87 (1.11)		
	Total sample (n=12)	3.91 (0.99)		
**The Illusionist**			13.5	.56
	Typically developing (n=7)	4.54 (0.35)		
	Disruptive behavior disorder (n=5)	4.04 (1.09)		
	Total sample (n=12)	4.33 (0.75)		
**Abaddon**			12.5	.42
	Typically developing (n=6)	4.13 (0.63)		
	Disruptive behavior disorder (n=6)	3.85 (0.45)		
	Total sample (n=12)	3.99 (0.54)		
**RegnaTools**			9.5	.20
	Typically developing (n=6)	4.08 (0.62)		
	Disruptive behavior disorder (n=6)	4.54 (0.45)		
	Total sample (n=12)	4.31 (0.57)		
**TimeOut!**			17.5	.94
	Typically developing (n=6)	4.31 (0.45)		
	Disruptive behavior disorder (n=6)	4.33 (0.66)		
	Total sample (n=12)	4.32 (0.54)		

Out of the 24 children who used mobile apps 5 and 6, 19 (79%) agreed/strongly agreed that they were more aware of themselves and their feelings, 23 (96%) agreed/strongly agreed that they had learnt skills to manage their anger, 22 (92%) agreed/strongly agreed that they wanted to learn more skills to manage their anger, 18 (75%) agreed/strongly agreed that they were motivated to use the skills learnt in the mobile apps in their daily lives, and 21 (88%) agreed/strongly agreed that the use of the mobile apps will help them manage their anger. Mann-Whitney tests indicated that there were no significant differences between the TD children and children with DBD in the perceived impact scores for mobile app 5 (RegnaTools), with *U*=14.5 and *P*=.62, and mobile app 6 (TimeOut!), with *U*=16.0 and *P*=.81.

### Aggression Levels

Wilcoxon signed rank tests indicated that children reported significantly lower reactive aggression after playing the mobile apps Rage Raver, with *Z*=−2.95 and *P*=.001; Abaddon, with *Z*=−2.52 and *P*=.008; and RegnaTools, with *Z*=−2.16 and *P*=.03. Children reported significantly lower overall aggression after playing the mobile apps Abaddon, with *Z*=−2.52 and *P*=.008 and RegnaTools, with *Z*=−2.14 and *P*=.03. There were no significant changes in proactive aggression in the children after the use of the mobile apps. The results are summarized in Table 8.

To understand if the use of the mobile apps affected the aggression scores of children with DBD differently from TD children, we analyzed the RPQ scores of these 2 groups of children separately for each mobile app, as shown in Table 9.

Wilcoxon signed rank tests showed that TD children who played the Rage Raver mobile app reported significantly lower reactive aggression after game play, with *Z*=−2.26 and *P*=.03. DBD children who used the RegnaTools mobile app reported significantly lower reactive and overall aggression after game play, with *Z*=−2.21, *P*=.03 and *Z*=−2.20, *P*=.03, respectively.

**Table 7:** Perceived impact scores.

Perceived impact	Typically developing (n=6), mean (SD)	Disruptive behavior disorder (n=6), mean (SD)	Total sample (n=12), mean (SD)
RegnaTools	4.33 (0.48)	4.47 (0.39)	4.40 (0.43)
TimeOut!	4.40 (0.64)	4.50 (0.64)	4.45 (0.62)

**Table 8:** Descriptive statistics and paired *Z* test analyses for Reactive-Proactive Aggression Questionnaire*.*

App name and aggression level			Before game, mean (SD)		After game, mean (SD)		*Z* value		*P* value
**Village of Lost Expressions (n=12)**									
	Reactive aggression	0.98 (0.40)		0.83 (0.43)		−2.0		.06	
	Proactive aggression	0.26 (0.22)		0.23 (0.23)		−0.49		.68	
	Overall aggression	0.60 (0.27)		0.52 (0.29)		−1.85		.07	
**Rage Raver (n=12)**									
	Reactive aggression	0.84 (0.49)		0.40 (0.27)		−2.95		.001	
	Proactive aggression	0.36 (0.42)		0.51 (0.37)		−1.49		.15	
	Overall aggression	0.59 (0.42)		0.46 (0.31)		−1.89		.07	
**The Illusionist (n=12)**									
	Reactive aggression	0.61 (0.41)		0.52 (0.48)		−1.22		.31	
	Proactive aggression	0.15 (0.28)		0.17 (0.23)		−0.94		.42	
	Overall aggression	0.37 (0.29)		0.34 (0.31)		−1.27		.23	
**Abaddon (n=10)**									
	Reactive aggression	0.90 (0.37)		0.65 (0.40)		−2.52		.008	
	Proactive aggression	0.18 (0.23)		0.15 (0.22)		−0.32		.88	
	Overall aggression	0.52 (0.24)		0.39 (0.26)		−2.52		.008	
**RegnaTools (n=12)**									
	Reactive aggression	0.70 (0.42)		0.53 (0.41)		−2.16		.03	
	Proactive aggression	0.23 (0.27)		0.19 (0.32)		−0.14		.98	
	Overall aggression	0.45 (0.28)		0.35 (0.29)		−2.14		.03	
**TimeOut! (n=12)**									
	Reactive aggression	0.79 (0.26)		0.70 (0.29)		−1.43		.17	
	Proactive aggression	0.12 (0.16)		0.08 (0.12)		−1.69		.10	
	Overall aggression	0.44 (0.19)		0.38 (0.18)		−0.85		.53	

**Table 9:** Descriptive statistics and paired *Z* test analyses for Reactive-Proactive Aggression Questionnaire according to condition*.*

App name and aggression level			Before game, mean (SD)	After game, mean (SD)	*Z* value	*P* value
**Village of Lost Expressions (n=12)**						
	**TD^a^** **(n=6)**					
		Reactive aggression	1.17 (0.46)	1.00 (0.55)	−1.29	.38
		Proactive aggression	0.28 (0.26)	0.19 (0.25)	−1.84	.13
		Overall aggression	0.70 (0.32)	0.58 (0.38)	−2.03	.06
	**DBD^b^** **(n=6)**					
		Reactive aggression	0.79 (0.23)	0.67 (0.21)	−1.60	.25
		Proactive aggression	0.24 (0.21)	0.26 (0.21)	−0.56	.75
		Overall aggression	0.50 (0.18)	0.46 (0.16)	−0.41	.81
**Rage Raver (n=12)**						
	**TD^a^** **(n=6)**					
		Reactive aggression	0.56 (0.07)	0.29 (0.19)	–2.26	.03
		Proactive aggression	0.08 (0.09)	0.35 (0.18)	–2.06	.06
		Overall aggression	0.31 (0.05)	0.32 (0.13)	0	.99
	**DBD^b^** **(n=6)**					
		Reactive aggression	1.12 (0.58)	0.52 (0.31)	−2.02	.06
		Proactive aggression	0.64 (0.44)	0.68 (0.45)	−0.41	.81
		Overall aggression	0.87 (0.44)	0.60 (0.38)	−2.02	.06
**The Illusionist (n=12)**						
	**TD^a^** **(n=7)**					
		Reactive aggression	0.56 (0.41)	0.51 (0.40)	−1.41	.50
		Proactive aggression	0.01 (0.03)	0.05 (0.09)	−0.82	.75
		Overall aggression	0.27 (0.21)	0.27 (0.22)	−0.37	.88
	**DBD^b^** **(n=5)**					
		Reactive aggression	0.67 (0.44)	0.53 (0.63)	−1.07	.50
		Proactive aggression	0.33 (0.38)	0.35 (0.25)	−0.37	.88
		Overall aggression	0.50 (0.37)	0.43 (0.42)	−1.07	.50
**Abaddon (n=10)**						
	**TD^a^** **(n=6)**					
		Reactive aggression	0.77 (0.35)	0.48 (0.36)	−2.02	.06
		Proactive aggression	0.11 (0.15)	0.01 (0.03)	−1.34	.50
		Overall aggression	0.43 (0.22)	0.24 (0.16)	−2.02	.06
	**DBD^b^** **(n=4)**					
		Reactive aggression	1.09 (0.34)	0.89 (0.37)	−1.60	.25
		Proactive aggression	0.27 (0.32)	0.35 (0.22)	−1.29	.38
		Overall aggression	0.66 (0.23)	0.61 (0.23)	−1.60	.25
**RegnaTools (n=12)**						
	**TD^a^** **(n=6)**					
		Reactive aggression	0.45 (0.34)	0.41 (0.46)	−0.55	.75
		Proactive aggression	0.15 (0.26)	0.06 (0.10)	−0.54	.75
		Overall aggression	0.30 (0.21)	0.22 (0.22)	−0.55	.75
	**DBD^b^** **(n=6)**					
		Reactive aggression	0.94 (0.37)	0.65 (0.35)	−2.21	.03
		Proactive aggression	0.31 (0.28)	0.32 (0.41)	−0.27	.94
		Overall aggression	0.61 (0.27)	0.48 (0.31)	−2.20	.03
**TimeOut! (n=12)**						
	**TD^a^** **(n=6)**					
		Reactive aggression	0.61 (0.09)	0.56 (0.18)	−0.74	.63
		Proactive aggression	0.03 (0.04)	0.03 (0.07)	−0.45	.99
		Overall aggression	0.30 (0.04)	0.28 (0.10)	−0.55	.75
	**DBD^b^** **(n=6)**					
		Reactive aggression	0.97 (0.25)	0.85 (0.31)	−1.09	.38
		Proactive aggression	0.21 (0.19)	0.14 (0.15)	−1.29	.38
		Overall aggression	0.57 (0.19)	0.48 (0.20)	−1.57	.16

^a^TD: typically developing.

^b^DBD: disruptive behavior disorder.

## Discussion

This study examined the usability and playability of RegnaTales, a series of 6 interactive mobile apps to address anger and aggression in children. All 6 RegnaTales mobile apps were perceived to be fun, appealing, and were able to sustain the interest of children, similar to what Ooi et al [31] found in their pilot study of first RegnaTales app on a nonclinical sample. Our study further demonstrated that both children with DBD and TD children reported high levels of enjoyment and playability in all 6 mobile apps. These findings are in line with existing literature that supports the use of serious games to deliver therapy or complement therapy in child and adolescent mental health [17,27,39]. However, the children in our study found mobile apps 1 and 3 (Village of Lost Expressions and The Illusionist) more challenging in comparison with other apps and required help in playing these 2 mobile apps. This highlights the need to investigate which aspects of the apps are perceived to be difficult (eg, words within the apps, complexity of instructions, gameplay difficulty, complexity of concepts taught) and indicate that adjustments to these 2 mobile apps are required to improve playability.

Mobile apps 5 and 6 (RegnaTools and TimeOut!) were designed as therapeutic tools to aid the user in the usage and application of anger management skills. Our results showed that most children were keen to continue using RegnaTools and TimeOut! after having used them once (with none of the children indicating *no* to continued usage). This is promising, given that 20% to 25% of users generally stop using a mobile app after the first use [40]. In addition, children who used RegnaTools and TimeOut! generally felt that the apps were helpful for them and rated being more aware of their feelings after using the mobile apps. It was heartening that 96% of them felt that they had learnt skills to manage their anger and 92% of them wanted to learn more skills to manage their anger, indicating a high level of motivation. This could possibly be a result of their positive experience learning these anger management skills in a fun and engaging manner. Crucial to the success of anger management is the application of the anger regulation skills taught in the person’s daily life to target behavioral change [41]. Our findings showed that most of the children were keen to apply the skills learnt from the mobile apps in their daily lives, and they generally felt optimistic that this would be helpful for them. The similarity in high perceived impact scores between children with DBD and TD children showed that RegnaTales was perceived to be helpful not only by children in the clinical population but also among normally developing children. Hence, RegnaTales has the potential to be used as a preventative tool, even before children start developing anger related problems.

Our findings showed that majority (81%) of the children were aware of the explicit goal of RegnaTales (ie, to save the hero’s parents), whereas a smaller percentage (58% to 71%) were aware of the implicit goals of the games (ie, to learn more about themselves and to learn to recognize other’s feelings). This indicates that we were somewhat successful in masking the true intent of the game, which is in line with the objective of a serious game that targets intrinsic motivation through fun [42]. This is important as Shen et al [43] have cautioned that the mere labelling of a game as educational could already elicit negative reactance in a player.

A secondary aim of the study was to ascertain through preliminary analysis, whether RegnaTales could be beneficial in reducing aggression. Although the study was designed as a playability and usability trial, and there was no control group, we observed a significant decrease in reactive and overall aggression measured immediately after game play in children when using some of the mobile apps. Reactive aggression is defensive, retaliatory, and is often a response to real or perceived threats or provocation. On the contrary, proactive aggression is displayed to attain a certain goal, which could be tangible or intangible. Reactive aggression is driven by the feelings of anger, defense, and retaliation, whereas proactive aggression is purposeful, deliberate, and goal-directed and is significantly less associated with anger. Our results indicated that reactive aggression was lower after the usage of Rage Raver, Abaddon, and RegnaTools. These apps were designed to help child users develop an awareness to their feelings, identify the link between emotions and thoughts (especially in the context of anger), as well as learn and practice adaptive anger regulation strategies (eg, deep breathing and cognitive restructuring). Hence the observed reduction in reactive aggression but not proactive aggression offers initial empirical support for the theoretical framework underlying RegnaTales. Furthermore, this finding is consistent with a previous systematic review that highlighted the feasibility of game-based learning in improving outcomes across diverse domains including psychopathology, behavior change, and acquisition of social skills [44]. Another pilot trial showed that children in an inpatient ward had less intense and less frequent anger episodes after a brief course of intervention on a biofeedback game platform based on CBT principles [45]. In our study, reductions in aggression scores were observed after the use of some, but not every, RegnaTales mobile app, despite the fact that many of the mobile apps shared similar content. The various mobile apps also appeared to have differing effects on aggression levels, depending on whether the children had DBD or not. However, these findings are only preliminary, given the small sample size of this study. Future studies with larger samples could shed light on the differential effectiveness of various components of this anger management program, and it would be fruitful to separately investigate their effects on children with DBD and TD children.

### Limitations

Some limitations should be taken into account when inferring these findings in relation to existing evidence. First, although our study yielded positive results in terms of the level of enjoyment, playability, and perceived impact of RegnaTales, its effectiveness as an anger management intervention has not been fully evaluated. Furthermore, rigorous evaluation is critical through future studies to fully determine whether RegnaTales would bring about improved outcomes in aggression. In our study, each child played through only 1 of the 6 mobile apps, which meant that each child was only exposed to a part of the anger management program. As RegnaTales was developed to be used as a complete program of 6 mobile apps, where anger management skills are progressively taught and built upon one another with a storyline that flows through, it would be more meaningful if each child were to play through all 6 mobile apps. This could yield differing results in terms of playability and changes in aggression scores. In addition, the findings presented should be regarded as preliminary and interpreted in consideration of the absence of a control group and the use of a pre- and postmethodological design. Another limitation is that our participants in the DBD group were all diagnosed with ADHD and only 1 had an additional diagnosis of ODD and 1 with an additional diagnosis of CD. Our DBD group is unlikely to be representative of the broader DBD spectrum, and it may be worthwhile to replicate the study with groups of children with ODD and CD to see if there are differing results. Future research where RegnaTales is being used and evaluated in the children’s natural environment (ie, at home and in school) instead of the clinic setting would be beneficial. Allowing the children to use the mobile apps at their own time and convenience and on their own mobile devices would yield a more accurate picture of the actual usage patterns of the mobile apps. This could also flag practical issues that may affect the usability of the mobile apps (eg, speed and stability of internet connectivity, using the mobile apps on mobile phones with much smaller screens).

### Conclusions

Our findings suggest that there is a potential for serious games in mental health interventions for children because of the games’ ability to engage and increase intrinsic motivation. RegnaTales is designed to be a serious game that is self-instructional and does not require explicit teaching or guidance. It is less manpower intensive as compared with face-to-face therapy and would be ideal in the context of soaring demand for mental health services for children amidst a mental health service that is growing at a much slower pace. This study provided positive preliminary findings, primarily in the domains of playability and usability of RegnaTales as a serious game. Coupled with future research to further determine its efficacy in reducing anger issues, RegnaTales could be a potential intervention for clinical as well as nonclinical populations.

## Appendix

Multimedia Appendix 1 Screenshots from the RegnaTales mobile apps.

## Abbreviations

ADHD: attention-deficit/hyperactivity disorder
CBT: cognitive behavioral therapy
CD: conduct disorder
DBD: disruptive behavior disorders
DSRB: Domain Specific Review Board
eSFQ: extended Short Feedback Questionnaire
ODD: oppositional defiant disorder
PUQ: Playability and Usability Questionnaire
RPQ: Reactive-Proactive Aggression
SPSST: Social Problem-Solving Skills Training
TD: typically developing
