# Supplementary material for: An Interactive Mobile App Game to Address Aggression (RegnaTales): Pilot Quantitative Study
Source: JMIR Serious Games. 2019 May 8;7(2):e13242. doi: 10.2196/13242 (PMC6530258; doi:10.2196/13242)
Supplement: Multimedia Appendix 1 [file games_v7i2e13242_app1.pdf]

Appendix 1

Screenshots from the RegnaTales Mobile Applications

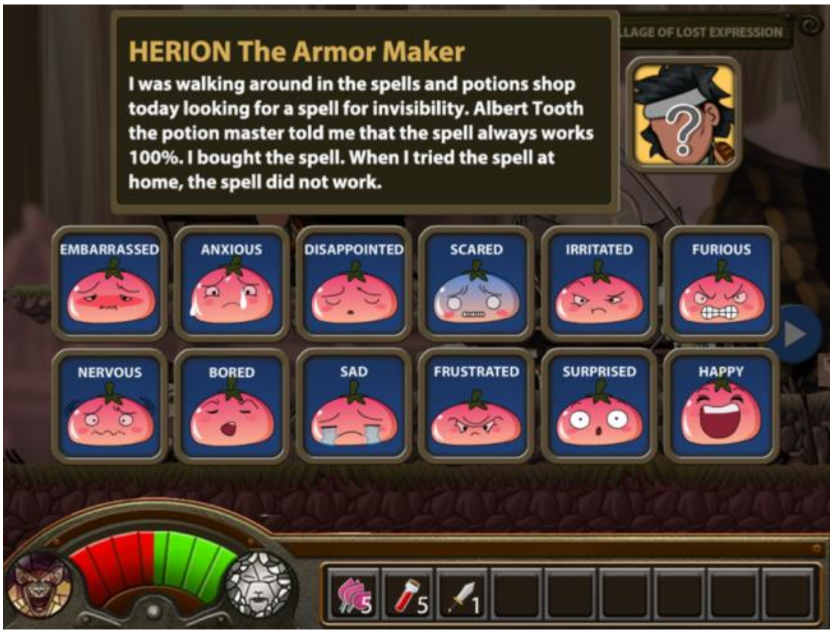

The player has to identify the feeling of the villager

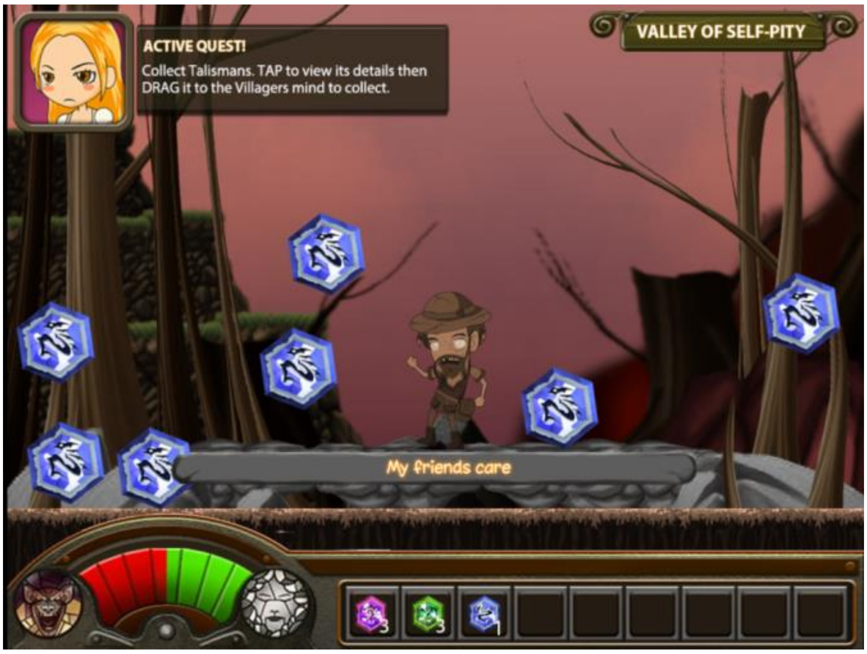

The player has to drag talismans containing positive and/or helpful thoughts to the villager's mind

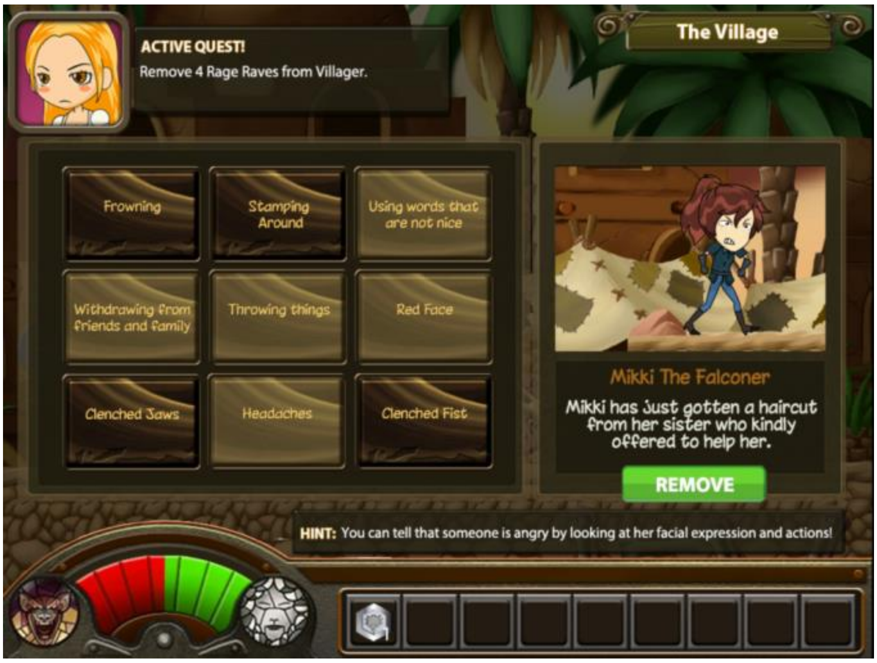

The player has to correctly identify the symptoms of anger

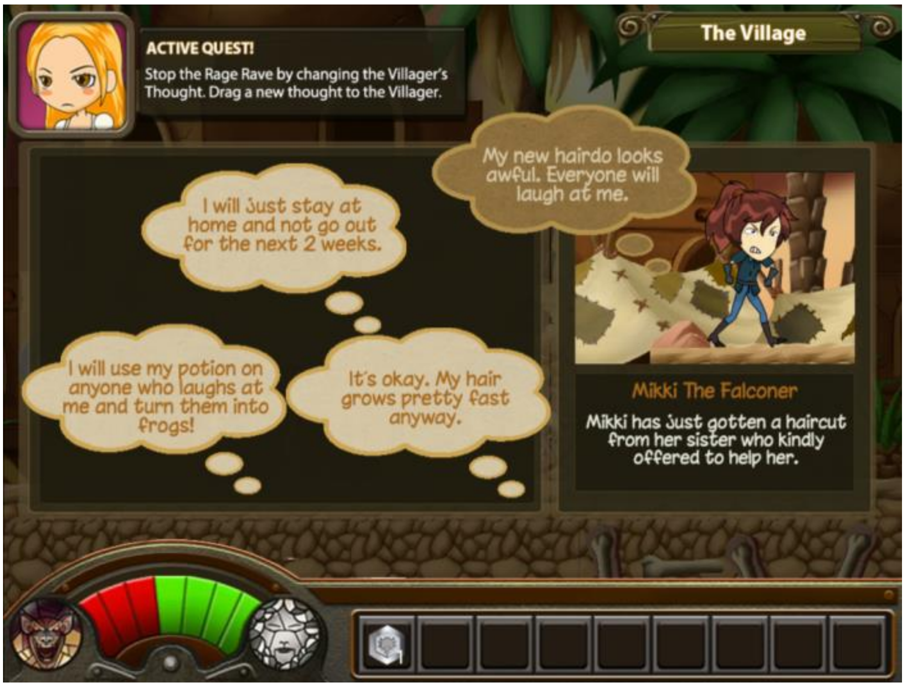

The villager has to replace the negative and/or unhelpful thought with a positive and/or helpful one

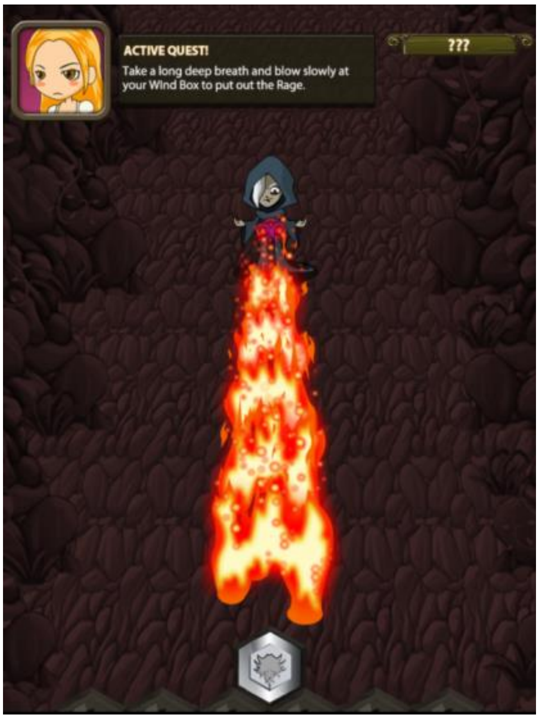

The player has to take deep breaths in order to extinguish the flame

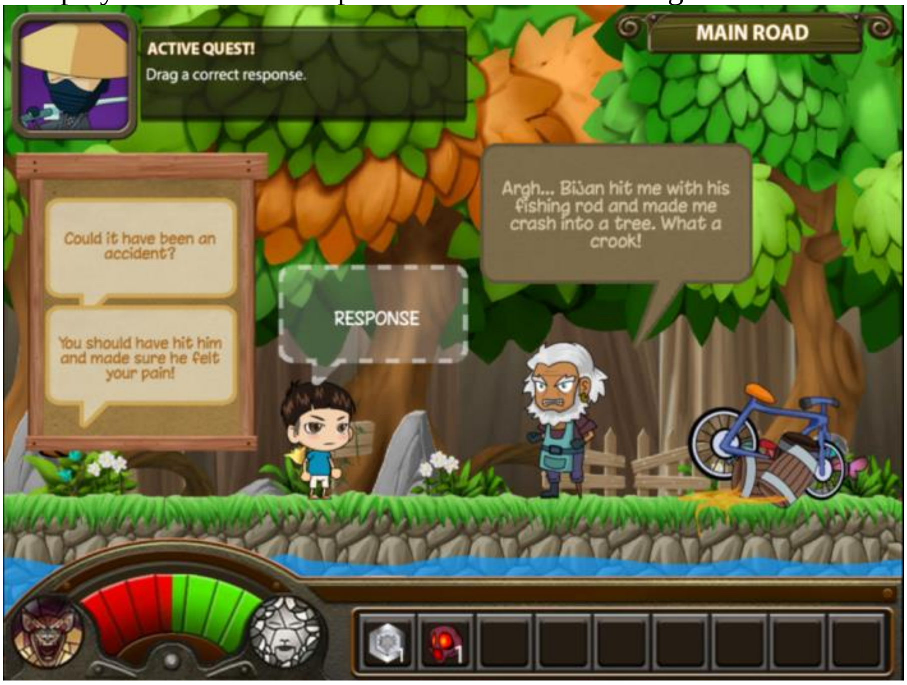

The player has to help the villager gain perspective taking

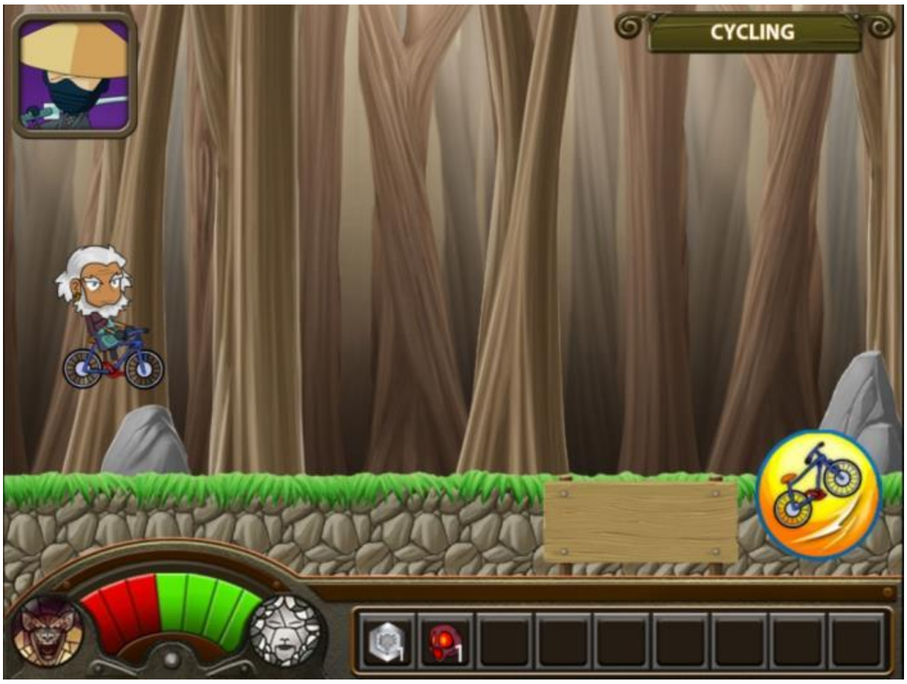

The player helps the villager deal with anger by engaging in pleasurable activities such as cycling

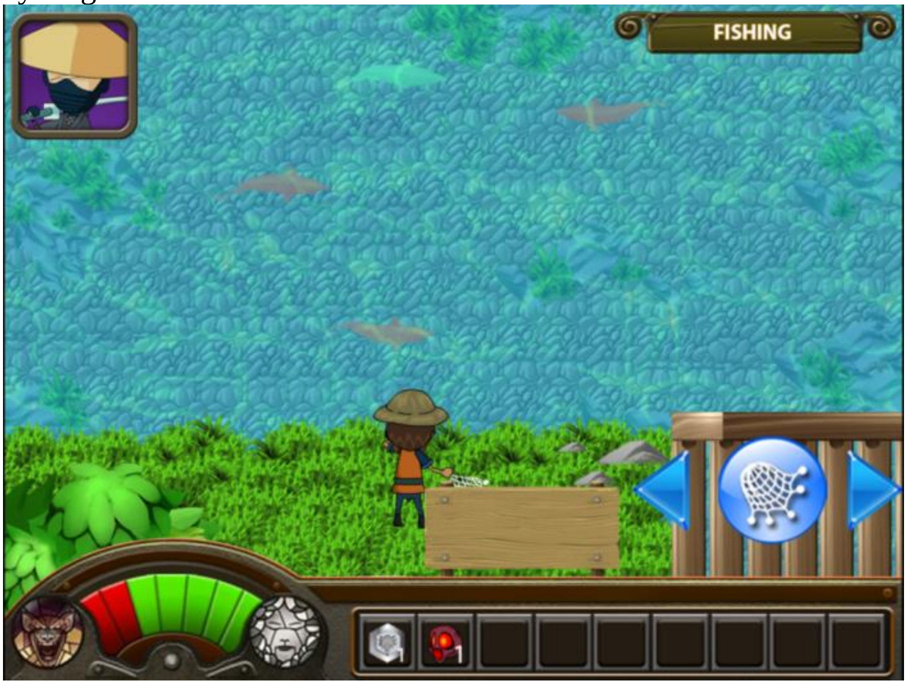

The player helps the villager deal with anger by engaging in pleasurable activities such as fishing

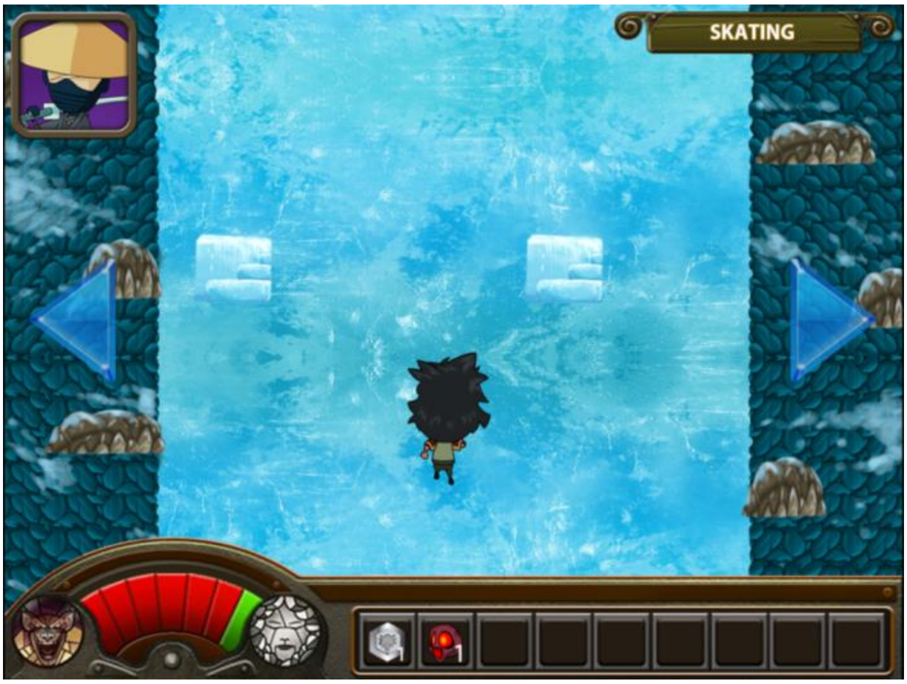

The player helps the villager deal with anger by engaging in pleasurable activities such as skating

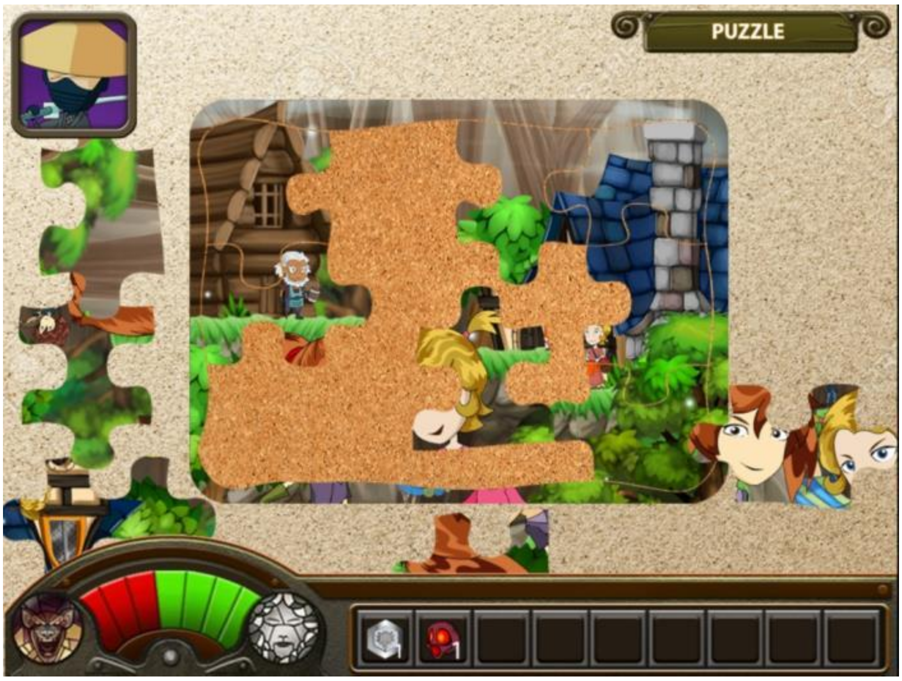

The player helps the villager deal with anger by engaging in pleasurable activities such as completing a jigsaw puzzle

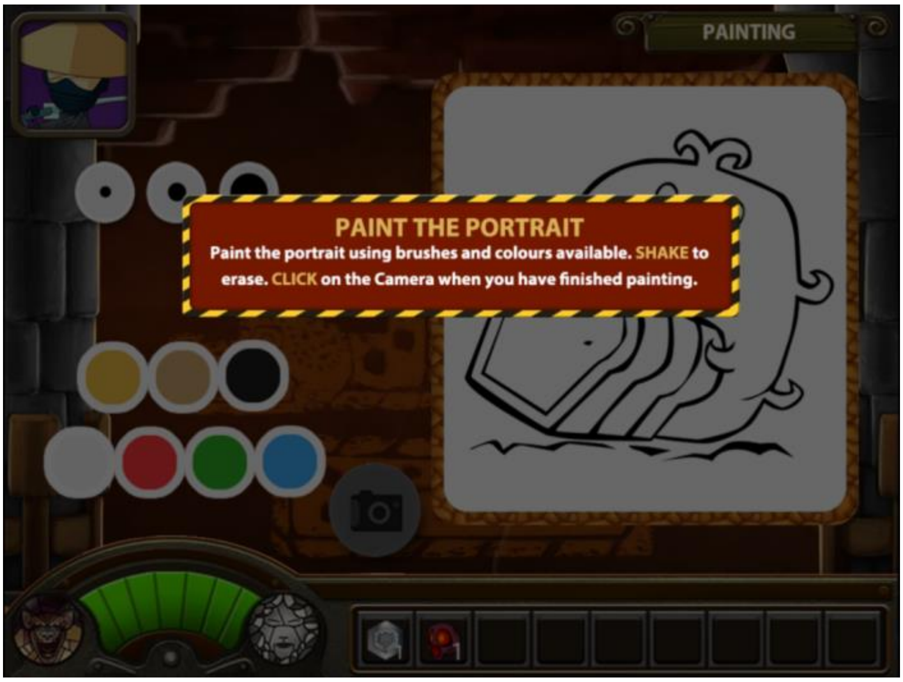

The player helps the villager deal with anger by engaging in pleasurable activities such as painting

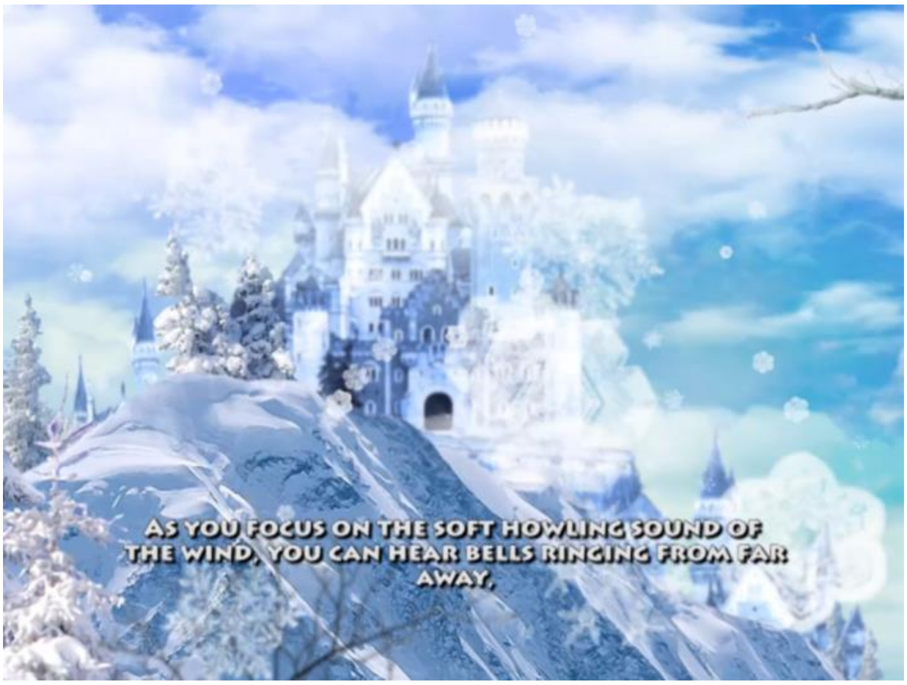

The player engages in guided imagery with visuals, sound effects, music and voice narration

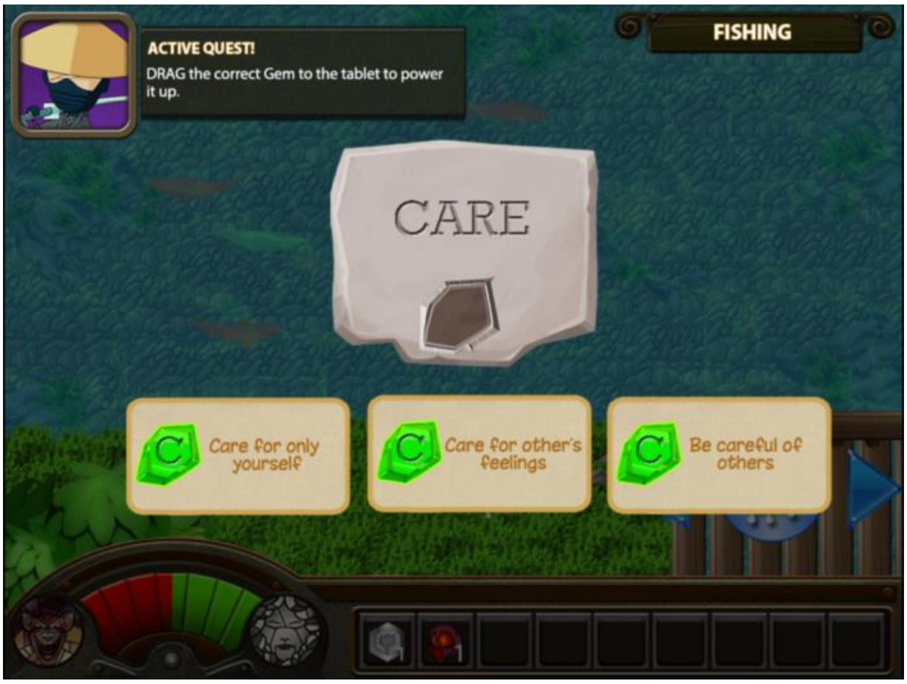

The player learns about Fighting Fair

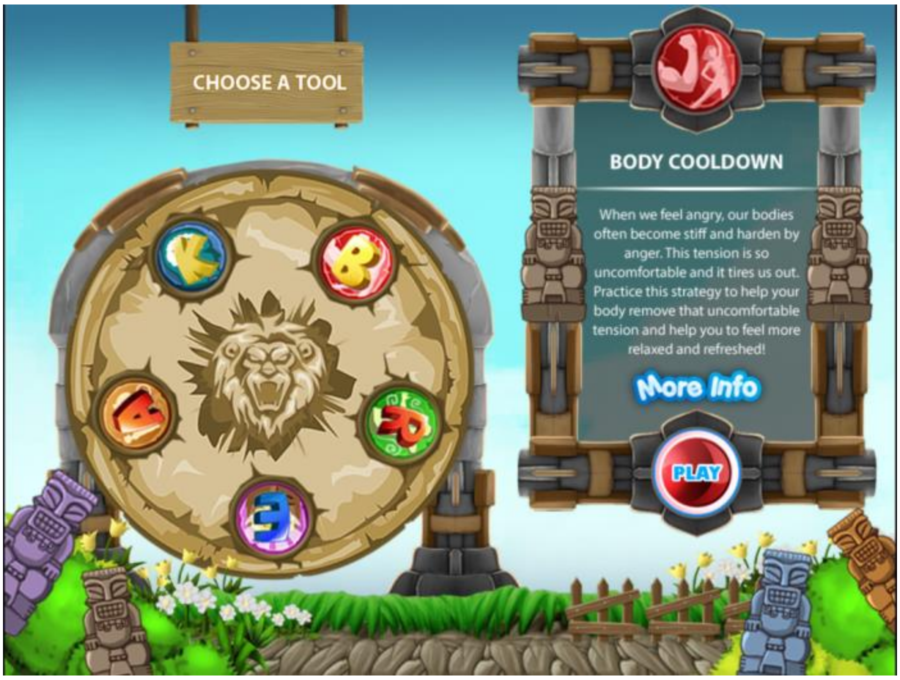

The player can choose to use a variety of anger coping activities in RegnaTools

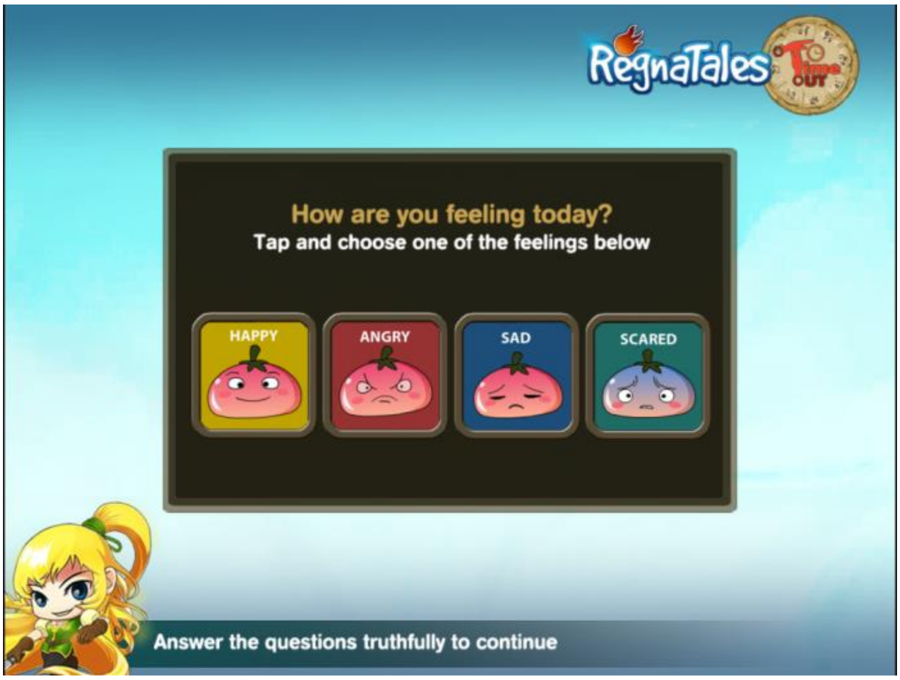

Player has to select his feeling on a daily basis in Timeout!

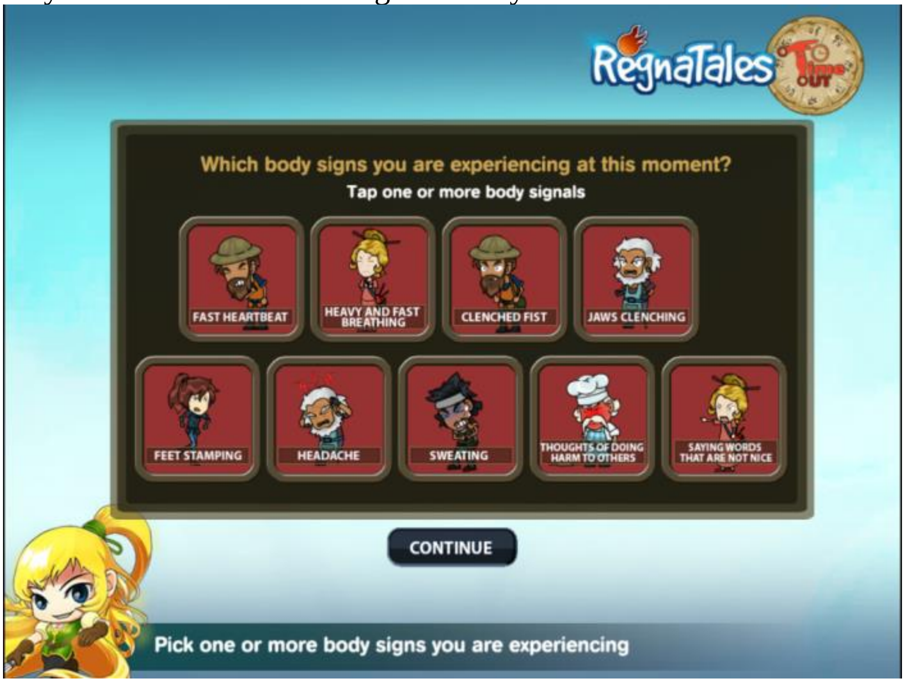

Player has to select his symptoms of anger in Timeout!

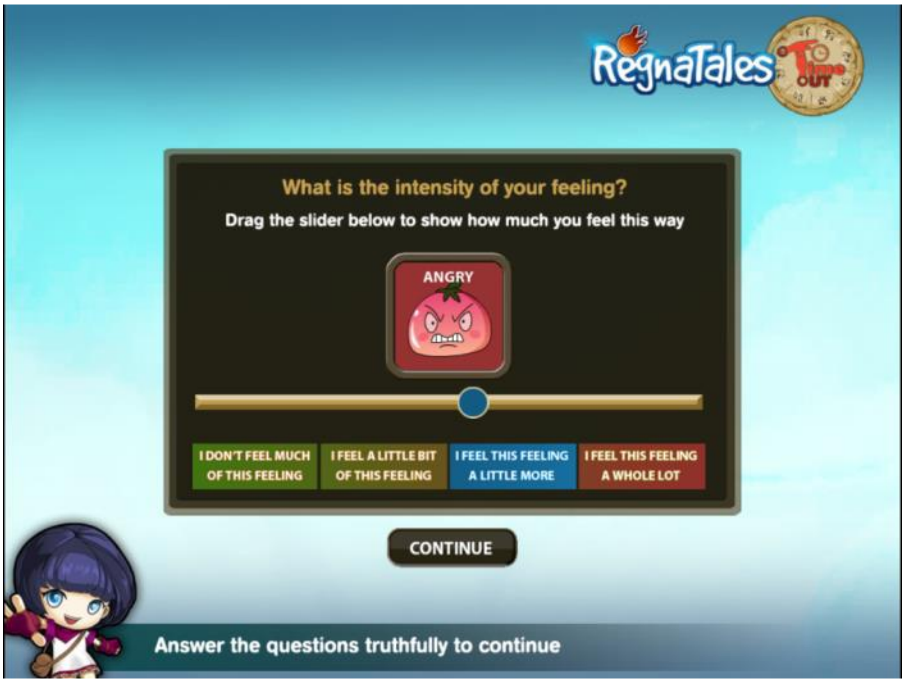

Player has to rate the intensity of his chosen feeling in Timeout!

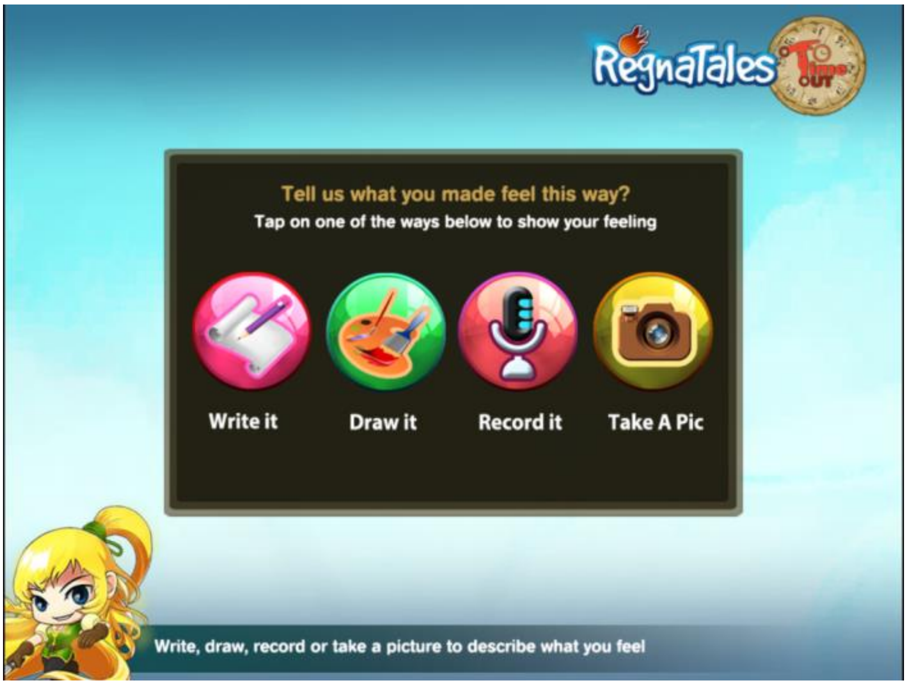

Player has to choose from a variety of methods to describe the trigger for the feeling in Timeout!

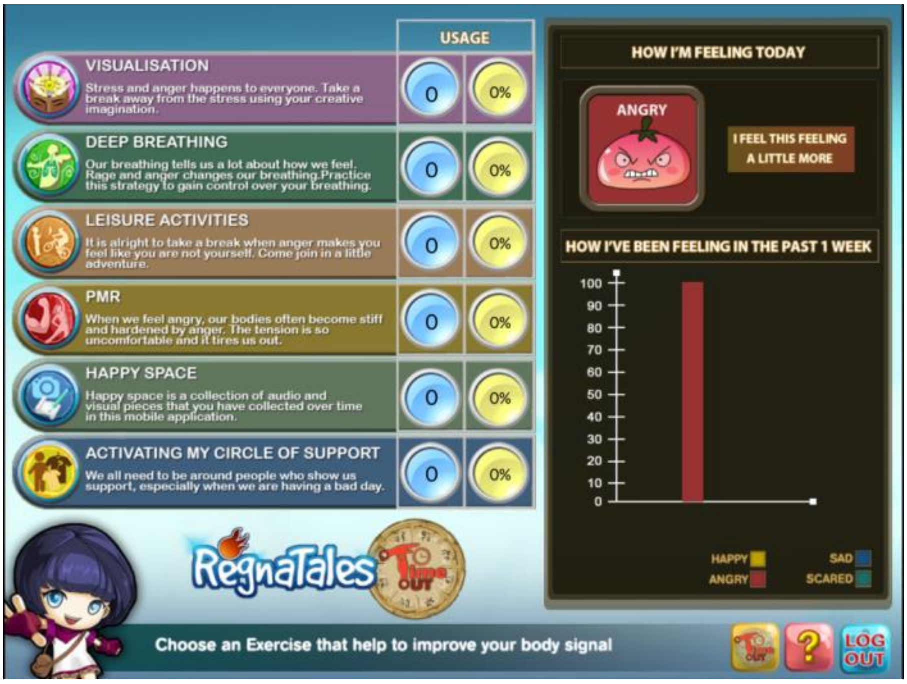

Player has to choose from a variety of strategies to cope with the negative feeling in Timeout!

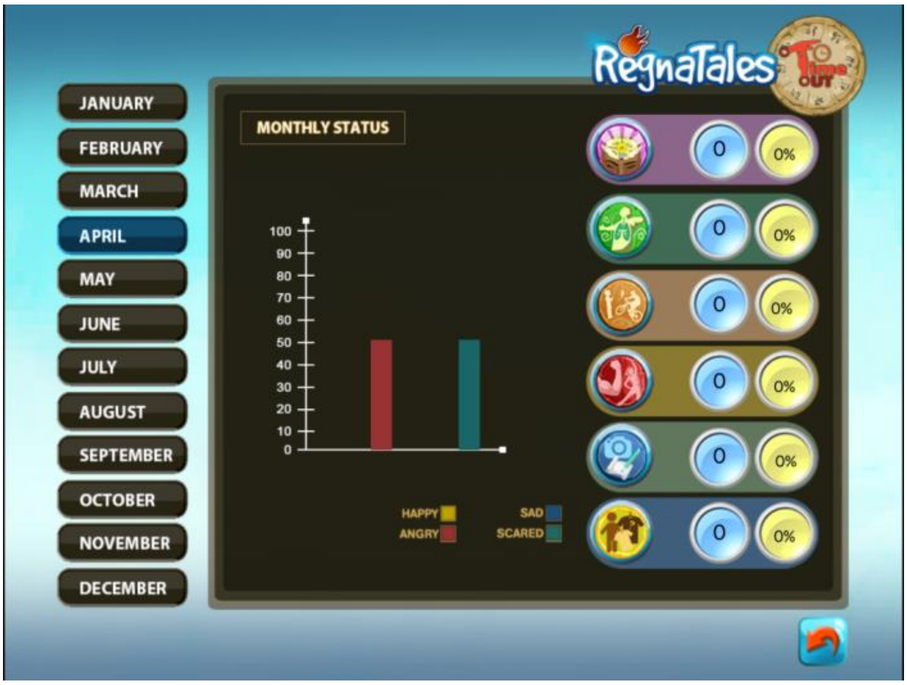

Player can view a chart on the frequency feelings and the usage of each anger coping strategy in Timeout!
